# *Nicotiana benthamiana* as a Production Platform for Artemisinin Precursors

**DOI:** 10.1371/journal.pone.0014222

**Published:** 2010-12-03

**Authors:** Teun W. J. M. van Herpen, Katarina Cankar, Marilise Nogueira, Dirk Bosch, Harro J. Bouwmeester, Jules Beekwilder

**Affiliations:** 1 Laboratory of Plant Physiology, Wageningen University, Wageningen, The Netherlands; 2 Plant Research International, Wageningen, The Netherlands; 3 Department of Chemistry, Utrecht University, Utrecht, The Netherlands; Purdue University, United States of America

## Abstract

**Background:**

Production of pharmaceuticals in plants provides an alternative for chemical synthesis, fermentation or natural sources. *Nicotiana benthamiana* is deployed at commercial scale for production of therapeutic proteins. Here the potential of this plant is explored for rapid production of precursors of artemisinin, a sesquiterpenoid compound that is used for malaria treatment.

**Methodology/Principal Findings:**

Biosynthetic genes leading to artemisinic acid, a precursor of artemisinin, were combined and expressed in *N. benthamiana* by agro-infiltration. The first committed precursor of artemisinin, amorpha-4,11-diene, was produced upon infiltration of a construct containing amorpha-4,11-diene synthase, accompanied by 3-hydroxy-3-methylglutaryl-CoA reductase and farnesyl diphosphate synthase. Amorpha-4,11-diene was detected both in extracts and in the headspace of the *N. benthamiana* leaves. When the amorphadiene oxidase CYP71AV1 was co-infiltrated with the amorphadiene-synthesizing construct, the amorpha-4,11-diene levels strongly decreased, suggesting it was oxidized. Surprisingly, no anticipated oxidation products, such as artemisinic acid, were detected upon GC-MS analysis. However, analysis of leaf extracts with a non-targeted metabolomics approach, using LC-QTOF-MS, revealed the presence of another compound, which was identified as artemisinic acid-12-β-diglucoside. This compound accumulated to 39.5 mg.kg^−1^ fwt. Apparently the product of the heterologous pathway that was introduced, artemisinic acid, is further metabolized efficiently by glycosyl transferases that are endogenous to *N. benthamiana*.

**Conclusion/Significance:**

This work shows that agroinfiltration of *N. bentamiana* can be used as a model to study the production of sesquiterpenoid pharmaceutical compounds. The interaction between the ectopically introduced pathway and the endogenous metabolism of the plant is discussed.

## Introduction

Malaria is the most deadly parasitic disease in the world. It is responsible for over 300 to 500 million cases and more than a million deaths each year, especially amongst children and pregnant women in Africa. During the 1960s, quinine-derived medicines such as chloroquinine were used in a campaign to eradicate malaria world wide. After initial success, gradually these medicines lost their efficacy, due to the development of drug resistance in the malaria parasite [1]. Consequently, new drug application strategies to treat malaria were required and the World Health Organization (WHO) in 2005 recommended the change to artemisinin-based combined therapy (ACT) as first-line malaria treatment [2].

**Figure 1:**
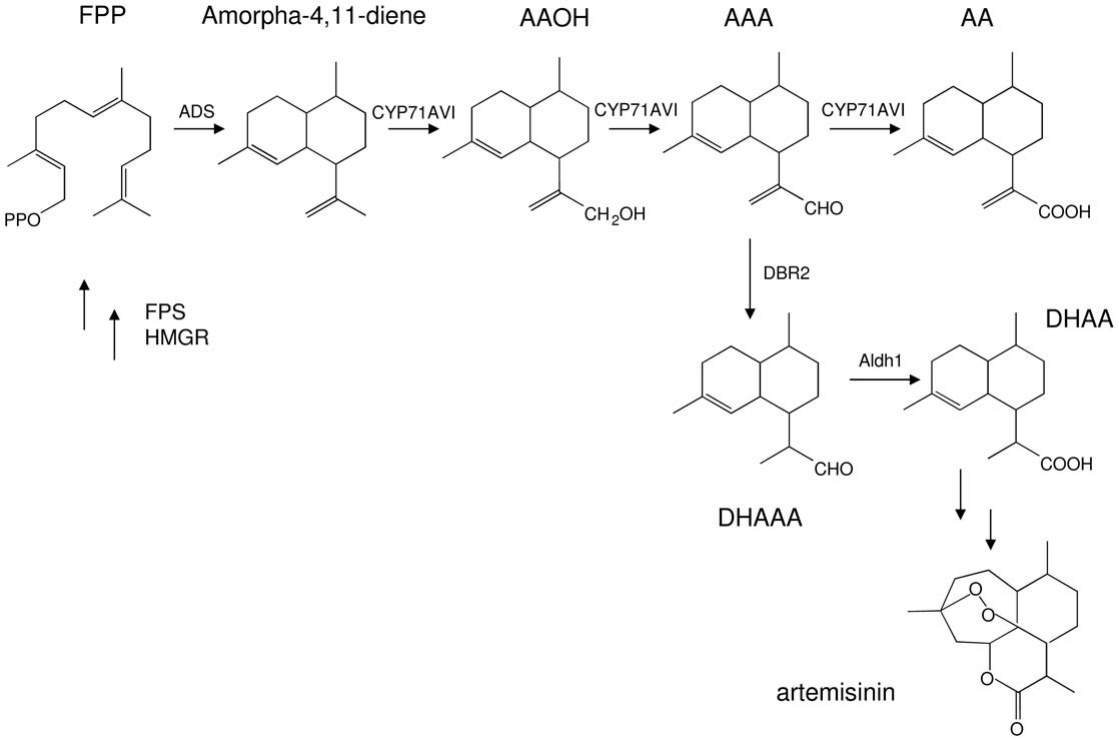
Biosynthetic pathway towards artemisinin. Genes relevant for this study have been indicated. ADS: amorpha-4,11-diene synthase; Aldh1: aldehyde dehydrogenase 1; CYP71AV1: amorpha-4,11-diene monooxygenase; DBR2: Artemisinic aldehyde double-bond reductase; FPS: farnesyl diphosphate synthase; HMGR: 3-hydroxy-3-methylglutaryl-CoA reductase; FPP: farnesyl diphosphate; AAOH: artemisinic alcohol; AAA: artemisinic aldehyde; AA: artemisinic acid; DHAAA: dihydro-artemisinic aldehyde; DHAA: dihydro-artemisinic acid.

Artemisinin has been discovered in the 1970s in China, as a highly effective against multidrug-resistant *Plasmodium spp*., the parasite that causes malaria [3]. It is a sesquiterpene lactone endoperoxide, which is extracted from *Artemisia annua L*, an herbaceous plant which is grown in China, Vietnam and eastern Africa. Artemisinin is produced in glandular trichomes, and its biosynthetic pathway has been elucidated ([4]; Fig. 1). The first committed step in the artemisinin pathway is mediated by amorphadiene synthase (ADS) [5] which converts farnesyl diphosphate (FPP) into amorpha-4,11-diene. Subsequently, a P450 enzyme (CYP71AV1) converts the amorpha-4,11-diene into artemisinic alcohol, artemisinic aldehyde and artemisinic acid [6], [7]. For production of dihydro-artemisinic acid, the most likely closest *in planta*-intermediate in artemisinin biosynthesis, a reductase (Dbr2) is needed [8], as well as a aldehyde dehydrogenase (Aldh1) [9].

Availability of artemisinin is limited by the low yield of *A. annua* and this is hindering availability of affordable malaria medicine to patients in developing countries. One approach to overcome this is the improvement of *A. annua* as a production system. A genetic map of *A. annua* has been published and genetic loci for artemisinin content have been identified [10]. Alternative strategies involve the production of artemisinin precursors in heterologous systems, such as micro-organisms. While total chemical synthesis of artemisinin is difficult and costly, the semi-synthesis of artemisinin from one of its precursors could be a cost-effective and reliable source of artemisinin. In search of alternative production systems, the engineering of *Saccharomyces cerevisiae* to produce artemisinic acid has been reported [6]. A large number of modifications, including ectopic expression of the mevalonate pathway and the *A. annua* genes for *ADS* and *CYP71AV1* resulted in production of artemisinic acid.

An agricultural crop with rapid accumulation of biomass at low costs could be an alternative for both *A. annua* and microbial-based production systems. *Nicotiana* spp have served as a host for the heterologous production of many medically relevant proteins using nuclear, chloroplast and transient transformation approaches (reviewed by [11]) and also for the production of amorphadiene. Introduction of amorphadiene synthase into *Nicotiana tabacum* (tobacco) resulted in the production of minute amounts of amorphadiene [5]. During the last five years, a number of potential technical improvements have become available. For example, the mitochondria of *Arabidopsis thaliana* have been shown to be a better environment for sesquiterpene synthase expression compared to the cytoplasma, probably due to a higher availability of farnesyl diphosphate in that compartment [12]. Likewise, others [13] have achieved significant improvement of sesquiterpene production by targeting the sesquiterpene synthases to the chloroplasts and co-expressing chloroplast-targeted farnesyl diphosphate synthase (FPS) and 3-hydroxy-3-methylglutaryl-CoA reductase (HMGR). The addition of the latter two genes increases the flux of sesquiterpene precursors to the sesquiterpene synthase: FPS supplies the direct precursor of amorphadiene, farnesyl diphosphate, while HMGR is considered the most important rate-limiting step in the mevalonate pathway [14], [15], [16]. This approach led to production levels of amorphadiene of up to 25 mg.kg^−1^ tobacco [13].

For the production of artemisinin precursors beyond amorphadiene, the introduction of even more than these three genes is required. When each gene is driven by the same promoter, often the repeated sequences may lead to silencing of the promoter or instability by recombination [17]. [13] avoided this by providing each gene with a different promoter. With increasing numbers of genes and hence promoters, however, the uncertainty that all these promoters are active in exactly the same cells increases. Another approach was described where two heterologous genes from the carotenoid biosynthesis pathway can be co-expressed in tobacco from a single promoter [18]. This is achieved by translating the enzymes from a single open reading frame, in which the individual proteins are separated by a viral peptide signal (2A). During translation, the 2A peptide leads to ribosomal skipping, and production of individual proteins. Recently it was described that, by using the ribosomal skipping technology, up to four genes from the glucosinolate pathway could be expressed from a single promoter in tobacco [19].

In the present study, we set out to produce artemisinic acid in *Nicotiana benthamiana*. To achieve this, multi-gene constructs with ribosomal skipping sequences and mitochondrial targeting sequences are used, and combinations of amorphadiene synthase, FPS, HMGR and CYP71AV1 are expressed. The results of this approach are evaluated by GC-MS, as well as by untargeted metabolomics using LC-QTOF-MS and NMR.

## Results

### Amorphadiene synthase fused to FPS and HMGR improves amorphadiene biosynthesis in infiltrated leaves

**Figure 2:**
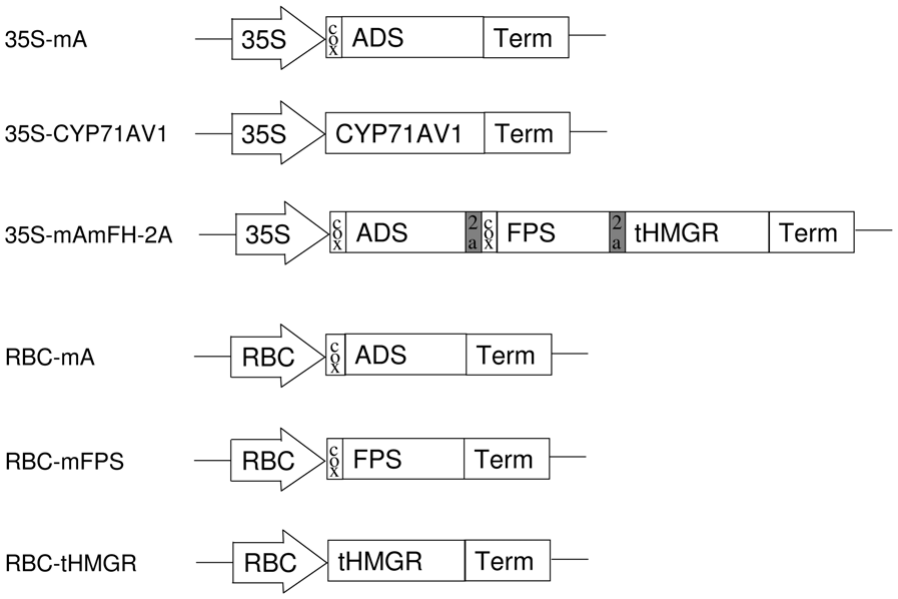
Constructs used for the different infiltration experiments. 35S: 35S CaMV promoter; cox: mitochondrial targeting signal; ADS: amorphadiene synthase; Term: Rubisco terminator; CYP71AV1: amorphadiene oxidase; FPS: farnesyl diphosphate synthase; tHMGR: truncated 3-hydroxy-3-methylglutaryl-CoA reductase. RBC: rubisco promoter.

To be able to express *ADS* in combination with *FPS* and *HMGR*, a construct was made in which these genes were in a single open reading frame, separated by 2A ribosomal skipping sequences (35S-mAmFH-2A) (Fig. 2). The effectiveness of this construct was compared with a single amorphadiene synthase (35S-mA).

Infiltrated *N. benthamiana* leaves were analyzed for emission of amorphadiene into their headspace. In the headspace of the 35S-mA and the 35S-mAmFH-2A plants, amorphadiene was detected while this was not observed for pBinPlus-infiltrated leaves. In the 35S-mA-infiltrated leaves, amorphadiene production was 0.47 mg.kg^−1^.24 h^−1^ whereas in the 35S-mAmFH-2A leaves production was 0.70 mg.kg^−1^.24 h^−1^ (Table 1). Even though amorphadiene production upon infiltration with the 35S-mAmFH-2A constructs was on average two-fold higher than with 35S-mA, due to the high variability in emission between individual leaves, the difference in the headspace was not significant (*P*>0.05).

**Table 1:** Production of different artemisinin precursors in *N. benthamiana* infiltrated with a range of different constructs.

	Headspace	Ethylacetate extract	Ethylacetate extract	Methanol extract
	amorphadiene(mg.kg^−1^ fwt.24 h^−1^)	amorphadiene(mg.kg^−1^ fwt)	unknown compound(mg.kg^−1^ fwt)^1^	artemisinic acid-12- β-diglucoside(mg.kg^−1^ fwt)
pBin^3^	0	0	0	0
35S-mA	0.47±0.22	0.9±0.2	5.6±2.6	0
35S-mAmFH-2A	0.70±0.34	6.2±0.6	20.8±1.6	0
35S-mAmFH-2A +35S-CYP71AV1	0.03±0.01	0.2±0.01	1.7±0.4	39.5±9.4
				
RBC-mA + pBin	nd^2^	0.011±0.009	0.006±0.004	nd
RBC-mA + RBC-mFPS	nd	0.04±0.009	0.06±0.01	nd
RBC-mA + RBC-tHMGR	nd	3.2±1.2	2.6±0.5	nd
RBC-mA + RBC-mFPS + RBC-tHMGR	nd	0.9±0.3	1.5±0.6	nd

1 concentration based on the response factor of amorphadiene.

2 nd  =  not determined;

3 Constructs are described in the text.

**Figure 3:**
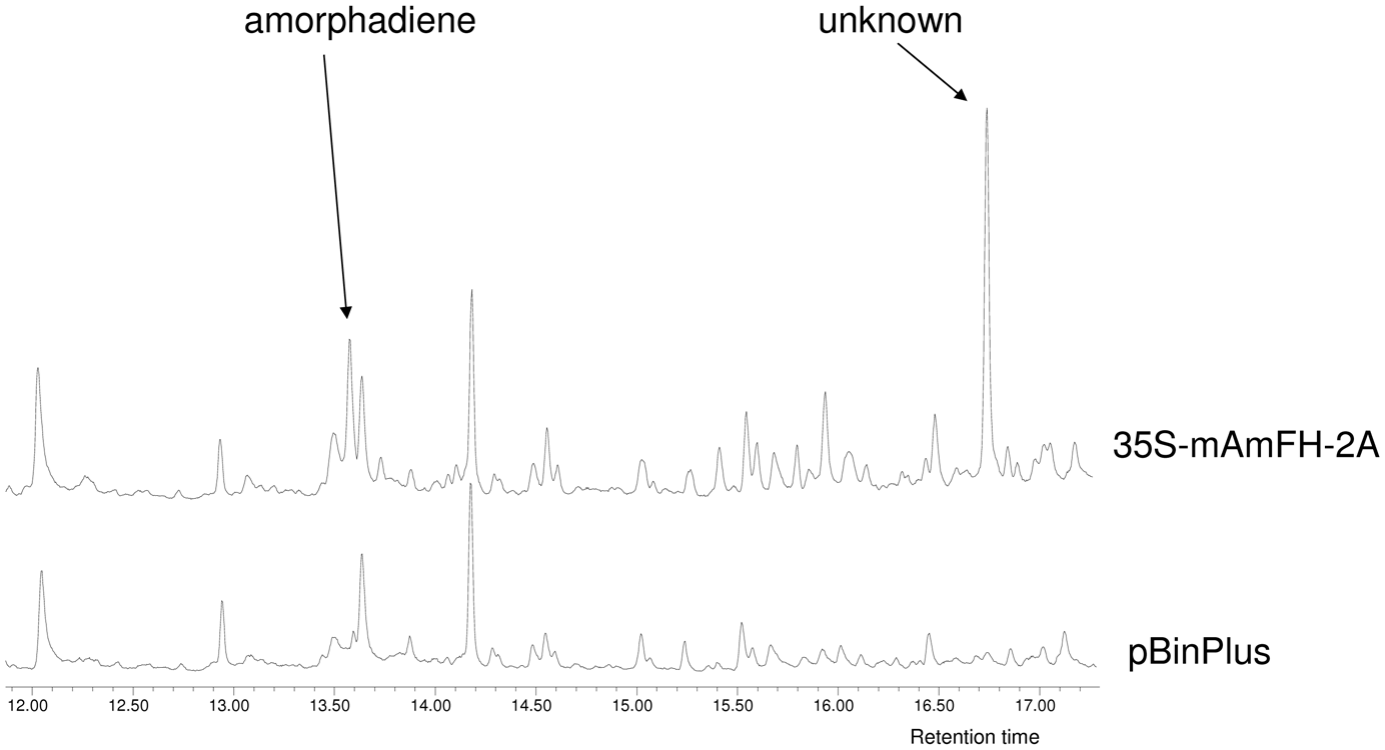
GC-MS chromatograms (total ion count) of ethyl acetate extracts of leaves infiltrated with 35S-mAmFH-2A (top) and a control construct (bottom).

In ethyl acetate extracts of the leaves, amorphadiene was also detected (Fig. 3). In addition to amorphadiene, a second, more dominant novel peak was detected at 16.8 min, in both 35S-mA and 35S-mAmFH-2A – infiltrated leaves, but not in control-infiltrated leaves. The identity of this compound remains to be established. Upon infiltration with the single gene 35S-mA construct 0.9 mg.kg^−1^ fwt amorphadiene and an estimated 5.6 mg.kg^−1^ fwt of the unknown compound were produced. Upon infiltration with the 35S-mAmFH-2A construct 6.2 mg.kg^−1^ fwt of amorphadiene and 20.8 mg.kg^−1^ fwt of the unknown compound were produced. Both amorphadiene and the unknown compound were significantly increased in the 35S-mAmFH-2A leaf extracts compared with leaves infiltrated with *ADS* alone (*P*<0.01).

Having established that the multi-gene 35S-mAmFH-2A construct resulted in the production of significantly more amorphadiene than the single-gene 35S-mA construct, we investigated what the contribution of *mFPS* and *tHMGR* was. Hereto, individual constructs RBC-mA, RBC-mFPS or RBC-tHMGR were used for co-infiltration experiments. In these experiments, gene expression was driven by the RBC promoter, instead of the 35S promoter and no P19 was co-infiltrated. Therefore, the results with these constructs can not be compared with the previous experiments using 35S-mAmFH-2A, but they can be used to assess the individual contribution of FPS and HMGR to the flux increase to amorphadiene. Infiltration of RBC-mA alone resulted in the production of 0.011 mg.kg^−1^ fwt of amorphadiene and 0.006 mg.kg^−1^ fwt of the unknown compound. Co-infiltration of RBC-mA with RBC-mFPS resulted in a relatively modest increase in the production of amorphadiene and the unknown compound to 0.04 mg.kg^−1^ fwt and 0.06 mg.kg^−1^ fwt, respectively. However, co-infiltration of RBC-mA with RBC-tHMGR resulted in a strong (300 to 400-fold) increase in accumulation of amorphadiene and the unknown compound (Table 1). Adding RBC-mFPS to the combination of RBC-mA and RBC-tHMGR did again not result in an increase in production. Apparently co-expression of in particular *tHMGR* has a strong contribution to amorphadiene biosynthesis in the mitochondria. The expression of *FPS* in the mitochondria, however, seems to be less rate-limiting.

### Amorphadiene is available for oxygenation by CYP71AV1

To further construct the pathway towards artemisinin in *N. benthamiana*, 35S-mAmFH-2A was co-infiltrated with a construct containing *A. annua CYP71AV1*. Co-expression of 35S-mAmFH-2A with 35S-CYP71AV1 caused a strong decrease in amorphadiene in the headspace compared with the 35S-mAmFH-2A construct alone (23-fold, from 0.70 to 0.03 mg.kg^−1^ fwt.24h^−1^; Table 1). Also in the ethylacetate extracts, both the amorphadiene and the unknown compound decreased strongly (31-fold from 6.2 to 0.2 mg.kg^−1^ fwt and 12-fold from 20.8 to 1.7 mg.kg^−1^ fwt, respectively; Table 1). Hence we assumed that amorphadiene was converted by CYP71AV1, for example to artemisinic alcohol (AAOH), artemisinic aldehyde (AAA) or artemisinic acid (AA) (Fig. 1). However, these metabolites were not observed in the GC-MS analysis, nor were any other novel metabolites detected, in either the headspace or the extract by GC-MS analysis.

**Figure 4:**
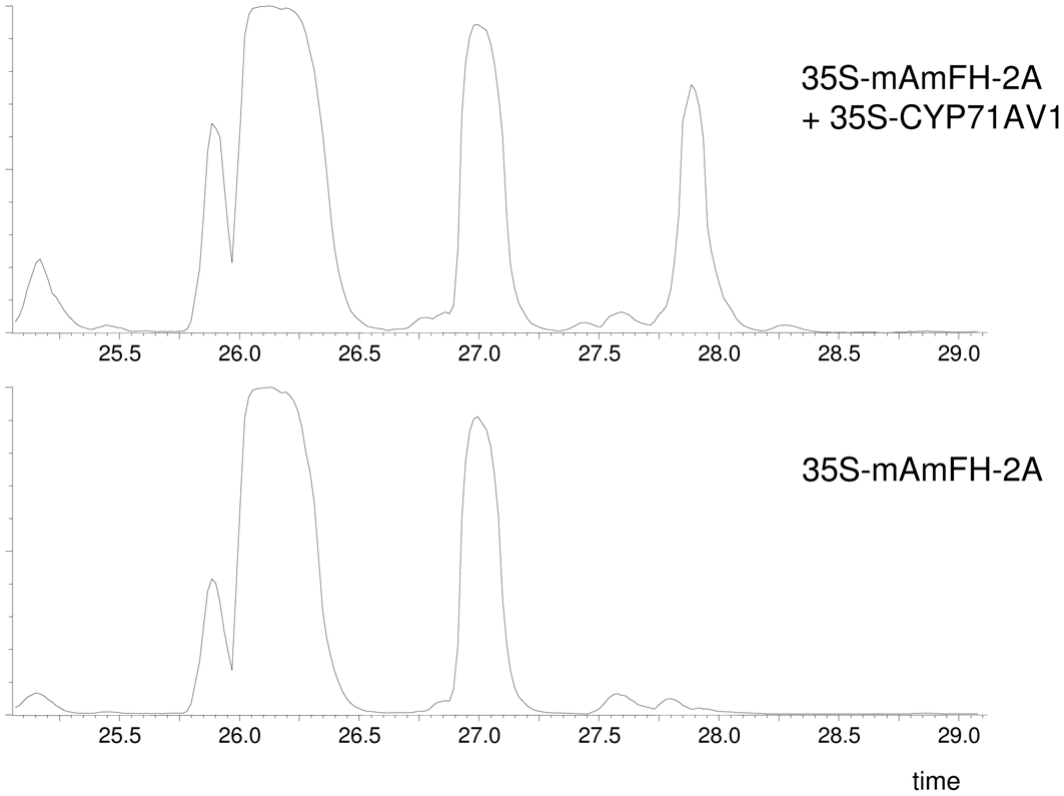
LC MS chromatograms (total ion count) of methanol extracts of *N. benthamiana* leaves, infiltrated with 35S-mAmFH-2A and 35S-CYP71AVI (top) and 35S-mAmFH-2Aand pBinPlus (bottom). Y-axis scale is identical in both chromatograms.

To investigate whether any other metabolic changes were caused by co-infiltration of *CYP71AV1*, an untargeted LC-QTOF-MS analysis of methanol extracts from leaves was carried out. Mass profiles of the pBinPlus, 35S-CYP71AV1, 35S-mAmFH-2A and 35S-mAmFH-2A +35S-CYP71AV1 samples were recorded and compared. The comparison of the 35S-mAmFH-2A +35S-CYP71AV1 co-infiltrated leaves to the mAmFH-2A infiltrated leaves revealed that, of the 6645 mass peaks taken into consideration, 63 mass peaks were increased >2-fold (P<0.01) due to co-infiltration of 35S-CYP71AV1 (Table S1). Fourty-eight of these mass peaks eluted at the same retention time of 28.0 min. Inspection of the mass chromatograms learned that indeed at 28.0 minutes, a clear peak was present in the 35S-mAmFH-2A +35S-CYP71AV1 extracts, which was absent in the control samples (Fig. 4). Further analysis of this peak by LC-MS showed dominant masses at *m/z* 1115.5190 ([2M-H2O-H]^−^) and at *m/z* 557.2548 ([M-H]^−^) (Fig. S1). LC-MS/MS of the base peak mass at *m/z* 557 showed fragments at *m/z* 395.2071 [M-Glc-H]-) and *m/z* 233.1562 ([M-2Glc-H]-) (Fig. S2). The presence of the 233.1562 mass peak suggested that artemisinic acid (calculated *m/z* = 233.1547) is part of this molecule, while the loss of 2 units of mass 162 suggests the presence of a di-C6-glycoside group. These observations lead us to hypothesize that the peak at 28.0 min in the LC-MS chromatograms represents an artemisinic acid-dihexoside.

### Identification of artemisinic acid-12-β-diglucoside

**Figure 5:**
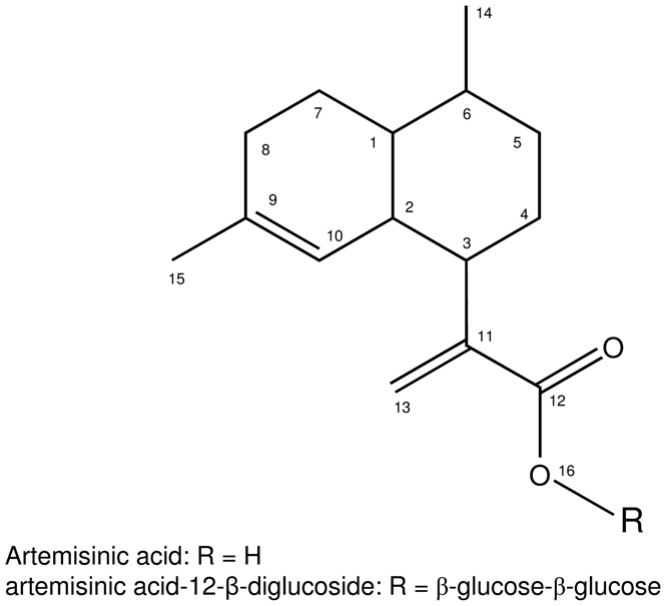
Structure of artemisinic acid (R  =  H) and artemisinic acid-12-β-diglucoside (R  =  β-glucose-β-glucose). Atom numbers are referred to in Table 2.

The putative artemisinic acid-dihexoside was purified by LC-SPE and subjected to 1H and 13C NMR. Comparison to an original standard of artemisinic acid was used to assign resonances to artemisinic acid (Table 2). A perfect overlap of resonances with artemisinic acid was observed, except that the the acetylene group of the artemisinic acid-moiety at C13 was slightly shifted compared to the artemisinic acid reference compound. Moreover the proton resonance of the OH of the carboxylic acid moiety at position 16 (the oxygen at carbon 12; Fig. 5) of the molecule, had disappeared. This supports coupling of the glycoside-moiety via the artemisinic acid acid function.

**Table 2:** ^1^H-NMR and ^13^C-NMR parameters of artemisinic acid and artemisinic acid-12-β-diglucoside.

artemisinic acid			artemisinic acid-12-β-diglucoside		
C13(ppm)	H1(ppm)	Atomnumber^b^	C13(ppm)	H1(ppm)	Atomnumber
28,4	1,39	1	28,8	1,40	1
38,9	2,51	2	38,4	2,48	2
43,2	2,64	3	42,8	2,7	3
26,7	1.39/1.32	4	27,3	1.39/1.32	4
36	1.70/1.06	5	35,8	1.71/1.08	5
42,5	1,37	6	42,1	1,40	6
26,9	1.90/1.77	7	26,9	1.94/1.77	7
26,4	1.94/1.52	8	25,8	1,52	8
135,8		9			
120,9	4,98	10	120,8	4,97	10
144,1		11			
168,6		12			
124,9	6.24/5.47	13	126,9	6.50/5.60	13
19,9	0,89	14	20	0,90	14
23,7	1,57	15	23,9	1,58	15
	9.10	16^a^			
			105	4,53	glucose-1
			93,7	5,63	glucose-1
			82,2	3,58	glucose
			77,9	3,38	glucose
			77,5	3,29	glucose
			77	3,18	glucose
			76,9	3,6	glucose
			75,4	3,1	glucose
			71,2	3,25	glucose
			70,4	3,37	glucose
			62	3,58	glucose-6
			61,8	3,72	glucose-6

a proton bound to oxygen in the carboxylic moiety of artemisinic acid.

b Atom numbers refer to Fig. 5.

The purified artemisinic acid-dihexoside compound was treated with different glycosidase enzymes and analyzed on LC-MS. Treatment with β-glucosidase showed a strong decrease in the *m/z* 557.3 peak at Rt = 28.0 min (representing artemisinic acid-diglucoside), appearance of a novel peak of *m/z* = 791.5 at Rt = 53.1 min (representing artemisinic acid-monoglucoside [2M–H]) (Fig. S3), and trace amounts of artemisinic acid in GC-MS analysis. No decrease in the *m/z* 557 peak, or appearance of the *m/z* 791 peak, could be observed after treatment with α-glucosidase or β-galactosidase, which lead us to conclude that the novel product is artemisinic acid-12-β-diglucoside.

### Quantification of artemisinic acid-12-β-diglucoside

To quantify the artemisinic acid-diglucoside produced, leaf extracts were first deglycosylated with a glucosidase mixture, Viscozym L. The deglycosylated artemisinic acid was then quantified by GC-MS. Consistent with the glycosidic nature of the artemisinic acid-diglucoside, Viscozym treatment resulted in the production of artemisinic acid (Fig. S4). The amount of artemisinic acid released by the treatment was 16.6 mg.kg^−1^ fwt, which corresponds to 39.5 mg.kg^−1^ fwt artemisinic acid-12-β-diglucoside (Table 1).

## Discussion

In this paper, production of a non-protein pharmaceutical in *N. benthamiana* is described. *N. benthamiana* can produce several precursors of the sesquiterpenoid antimalarial drug artemisinin. Amorphadiene production was strongly elevated relative to experiments reported earlier by us [5]. Two novel strategies were used: firstly, amorphadiene synthase was targeted to the mitochondria, and secondly, mitochondrial *FPS* and cytosolic *HMGR* were co-expressed using a ribosomal skipping construct. When this gene combination was co-infiltrated with amorphadiene oxidase a novel compound identified as artemisinic acid-12-β-diglucoside was produced at 39.5 mg.kg^−1^ fwt. Thereby, this research adds to the potential of *N. benthamiana* as a model for production of plant-made pharmaceuticals. So far, *N. benthamiana* has been successfully explored as a model for expression of therapeutic proteins, such as antibodies [20]. To achieve this, a number of quick and efficient methods have been developed for *N. benthamiana*, including agro-infiltration and viral vectors. More recently, *N. benthamiana* agro-infiltration experiments have also been used to reconstruct the glucosinolate biosynthesis pathway [21]. In the present paper, agro-infiltration of *N. benthamiana* is deployed to rapidly screen engineering concepts for sesquiterpene production, and to investigate the interaction of the sesquiterpene product with endogenous metabolism of the host.

### Emission, accumulation and modification of amorphadiene

Production of sesquiterpenes in transgenic plants has so far mostly been investigated by extraction of leaf material with organic solvents [5], [13]. For research on plant-insect interaction, the emission of ectopic sesquiterpenes into the headspace has been described [12], [22]. Our headspace trapping experiments on *N. benthamiana* leaves show that part of the amorphadiene that is produced is emitted. After 10 days the concentration of amorphadiene in leaves infiltrated with the 35S-mA construct (0.9 mg.kg^−1^ fwt amorphadiene) is in the same order of magnitude as the emitted amorpha-4,11-diene (0.47 mg.kg^−1^ fwt.24 h^−1^). When multiple days of emission are taken into account, the emitted amount is probably significantly higher than the accumulated amount.

In the present paper, the sesquiterpene synthase was targeted to the mitochondria, using the COX targeting sequence, instead of the cytosol, where sesquiterpene biosynthesis takes place *in planta*. A farnesyl diphosphate synthase with mitochondrial targeting has been identified in Arabidopsis [23] and it has been shown that fusion of nerolidol synthase from strawberry to the COX targeting sequence supports biosynthesis of nerolidol (emitted into the headspace of transgenic Arabidopsis plants) whereas this was not achieved using cytosolic nerolidol synthase [12]. This is in keeping with our observation that amorphadiene is readily observed in extracts from *N. benthamiana* infiltrated with 35S-mA (0.9 mg.kg^−1^), while cytosolic production of amorphadiene in stable *N. tabacum* tranformants occurred at much lower levels (0.2 to 1.7 µg.kg^−1^; [5]).

Production of amorphadiene was boosted by fusing *ADS*, through ribosomal skipping sequences, to *FPS* and *tHMGR*. Although emitted amorphadiene hardly increased (from 0.47 to 0.70 mg.kg^−1^ fwt.24 h^−1^) the concentration of amorphadiene accumulated in the leaf strongly increased (from 0.9 to 6.2 mg.kg^−1^ fwt). Apparently, the emission of amorphadiene becomes limiting when biosynthesis increases resulting in increased accumulation in the plant. It is unclear whether *N. benthamiana* stores the heterologous sesquiterpenes in specific organs, such as glandular trichomes (and from which evaporation then also occurs), and whether the 35S-promoter mediates expression in these organs. Alternatively, amorphadiene could be directly emitted from the mesophyl cells ([24] and refs therein). Very little is known about the mechanisms involved in volatile emission from *Nicotiana* leaves, and this aspect should be further investigated, for example using dynamic headspace trapping methods.

The strong increase in the production of amorphadiene by the use of a translational fusion of *ADS* to *FPS* and *tHMGR* could have several reasons. Overexpression of *tHMGR* has been shown to boost sesquiterpene expression [13], [15]. Likewise, co-expression of *FPS* with sesquiterpene synthases in the chloroplast compartment improved sesquiterpene expression [13]. In the present work *ADS* and *FPS* were co-expressed in the mitochondria. Co-infiltration with single-gene constructs containing *tHMGR* and *ADS* shows that *tHMGR*, which is expressed without targeting signal, strongly enhances amorphadiene production, much more than co-expression of mitochondrial targeted *FPS* (Table 1). Apparently, HMGR-produced isopentenyl diphosphate (IPP) is also available to the mitochondria and not only to the plastids as was demonstrated [13]. Also, the transport capacity to the mitochondria for IPP is apparently not limiting, or it may be up-regulated upon increased substrate demand. A second advantage of the 2A-coupled *ADS-FPS-HMGR* construct could be the coordinated expression of each of the subunits. When individual integrations into the genome are used for each of the subunits, genes that are part of the same pathway may not be expressed to the same level or even in the same cell, even when expressed from identical promoters. The latter is illustrated by experiments showing that different integration events of 35S-luciferase constructs lead to poorly overlapping expression patterns [25]. Obviously, when *HMGR*, *FPS* and *ADS* would not be expressed in the same cells, the effect of stimulating the flux into the terpene production pathway would be lost. A potential disadvantage of the 2A system could be that proteins downstream in the triple-gene open reading frame may have a reduced expression [19]. Therefore careful tuning of the order of genes may be needed to achieve optimal sesquiterpene production.

### Coexpression of a cytochrome P450 leads to accumulation of artemisinic acid di-hexoside

The advantage of a plant host for the production of sesquiterpenoids is their ability to accommodate P450 enzymes. In prokaryotic organisms, the use of specific, plant-derived P450s requires extensive engineering [6]. Therefore yeast, which also has a membrane system, has often been used as a model system for plant P450 expression (Olry et al., 2007; Urban et al., 1997). However, full conversion of sesquiterpene substrates in yeast requires extensive optimization of the genetics and growth conditions [6]. The work of Lucker et al. [26] shows that the plant P450 limonene-3-hydroxylase, when expressed in tobacco together with limonene synthase, readily converts a significant amount of the monoterpene limonene into (+)-*trans*-isopiperitenol. Here we show that CYP71AV1, a sesquiterpene oxidizing P450, also functions efficiently in *N. benthamiana*, and leads to almost complete conversion of amorphadiene. Interestingly, the intermediates in the oxidation of amorphadiene to artemisinic acid, artemisinic alcohol and artemisinic aldehyde, were not detectable in our experiments. This possibly reflects an efficient catalytic environment for CYP71AV1 when expressed in *N. benthamiana*.

*N. benthamiana* stores artemisinic acid principally as artemisinic acid-diglucoside. Apparently, glycosyl transferases of *N. benthamiana* – constitutively active or induced upon introduction of these transgenic metabolites - are very efficient in catalyzing this conversion, since hardly any other metabolite of amorphadiene could be observed. *N. tabacum* is known to express, upon pathogen infection, a UDP-glucosyl transferase *TOGT1* that glycosylates the acid moiety of phenolic compounds such as salicylic acid and coumaric acid [27]. The homologue of this enzyme in *N. benthamiana* may be the enzyme that initiates the modification of artemisinic acid to the diglucoside. The diglucoside is likely stored into the vacuole by a glucoside specific H^+^-coupled transporter, like has been shown for other phenolic glucosides [28]. Storage of target metabolites as glycoside may have the additional advantage that the storage capacity of the vacuole is used and that high concentrations can be achieved without phytotoxic effects. Glycosides can be extracted relatively easily from plants by hydrophylic solvents, and, after hydrolysis, the desired aglycon can be conveniently recovered at high concentrations in an organic phase.

The interaction between endogenous metabolism and products of ectopically expressed genes, as we observe here with the production of artemisinic acid di-glucoside, is a highly significant aspect of plant metabolic engineering. Often this aspect is ignored, because the applied detection methods are focused on finding the anticipated product. By use of a broad spectrum of unbiased analytical methods such as LC-MS, GC-MS of both extracts and headspace, and statistical data analysis tools, the role of metabolic cross-talk between endogenous and ectopic pathways can be uncovered. Metabolic cross-talk as a result of plant metabolic engineering is a phenomenon that has been scarcely described. Nevertheless, it may offer novel products with potential new biological activities, as has been shown for yeast [29], and as we show here for plants.

### Opportunities to increase artemisinic acid-productivity of *N. benthamiana*

The application of *N. benthamiana* agroinfiltration is a novel and highly effective way for producing sesquiterpenoids. In 10 days, infiltrated *N. benthamiana* leaves accumulated up to 6 mg.kg^−1^ fwt amorphadiene, depending on the construct used, in addition to an emission of amorphadiene at 0.5 mg.kg^−1^ fwt.24 h^−1^, and accumulation of an amorphadiene-related compound to an estimated amount of 20 mg.kg^−1^ fwt. This is a significant improvement relative to the levels of amorphadiene (0.2 to 1.7 µg.kg^−1^ fwt) that were obtained earlier using *ADS* alone in transgenic *N. tabacum* [5]. Later, strong improvement was achieved by the co-expression of *ADS* and *FPS* with chloroplast targeting in combination with un-targeted *tHMGR* resulting in an amorphadiene production of 25 mg.kg^−1^ fwt [13]. Using *N. benthamiana* agroinfiltration we managed to produce concentrations that are in the same order of magnitude as in the latter paper in only 10 days. In the present paper, artemisinic acid was produced to 16.6 mg.kg^−1^ fwt (as 39.5 mg.kg^−1^ fwt artemisinic acid diglucoside) in *N. benthamiana* by agroinfiltration. This compound has not yet been produced in stable transgenic tobacco.

The conversion of amorphadiene towards artemisinic acid diglucoside is quite efficient. The amount of amorphadiene found in the extracts of plants infiltrated with 35S-mAmFH-2A (6.2 mg.kg^−1^) is lower than is found as artemisinic acid-12-β-diglucoside in the presence of CYP71AVI (16.6 mg.kg^−1^ artemisinic acid; Table 1). The excess of oxygenated amorphadiene could derive from emitted amorphadiene, and/or from the amorphadiene-related unknown compound, for which the identity could not be established. Both of these potential sources are dramatically reduced upon co-infiltration (Table 1), suggesting that also (precursors of) the unknown compound is turned over by CYP71AVI. The potential capacity of amorphadiene (including the unknown compound) was estimated at 27.0 mg.kg^−1^ (Table 1), though it should be noted that the response-factor of the unknown compound is not known. The loss of ±10 mg.kg^−1^ amorphadiene-related compounds could result from the fact that the co-infiltration of CYP71AVI causes a reduction in ADS production, due to dilution of the *Agrobacterium* expressing ADS. Alternatively, a fraction of the amorphadiene may have been metabolized into other compounds. However, other compounds, except for the unknown compound, were hardly found in the LC-MS (Table S1) or in the GC-MS analysis (not shown).

*N. benthamiana* leaves are difficult to compare to yeast cultures, where artemisinic acid has been produced to a level of 100 mg.L^−1^ [6]. Though fermentation of microbes to produce plant compounds has strongly developed over the last 5 years, plants offer a number of benefits [30]. These include low startup costs, high flexibility in terms of scale and storage, and the potential to produce high product volumes at relatively low cost. We feel that *N. benthamiana* deserves further study to develop it as a production host. The agro-infiltration system used in this study is suitable for functional testing of genes and constructs, but relatively inefficient for large-scale metabolite production. Analysis by SDS PAGE of leaves subjected to agro-infiltration with *mADS* did not reveal detectable ADS protein (data not shown). However, [31] report accumulation of recombinant proteins to up to 10% of soluble protein in *N. benthamiana*, in 10 days, using a plant virus-based modular expression vector. Such vector optimization steps, in addition to careful balancing of *HMGR*, *ADS* and *CYP71AV1* expression, may still need to be made in *N. benthamiana*, before competitive production of artemisinic acid-derivatives can be achieved. For practical application, stable transformation of *N. benthamiana*, or likely other plant species, is probably required to reach commercially attractive high yields comparable to *Artemisia annua* or engineered micro-organisms.

### Conclusions

This work shows that agroinfiltration of *N. bentamiana* can be used as a model to study the production of sesquiterpenoid pharmaceutical compounds. By using mitochondrial targeting and 2A technology, novel compounds, formed by metabolic crosstalk between the endogenous metabolism and the ectopically expressed sesquiterpene biosynthetic pathway, can be produced. By using an untargeted metabolomics approach, and analysis of the headspace, organic extracts and semipolar extracts, such novel metabolites can be uncovered.

## Material and Methods

### Single constructs and 2A construct (mAmFH-2A)

An overview of the constructs used in this study is provided in Fig. 2. PCRs were performed with Phusion enzyme (Finnzymes, Finland). All the plasmids were constructed using standard cloning methods (restriction and ligation). The final and intermediate vectors were checked by restriction and sequencing. The 35S-mA vector was created by amplifying the *ADS* cDNA using AdsF and AdsR primers (Table 3) introducing a BamHI and a NotI restriction site. The restricted PCR product was introduced in the pIV2A_2.5 vector containing a CaMV35S promoter, CoxIV mitochondrial targeting sequence and a RbcS1 terminator (www.pri.wur.nl/UK/products/ImpactVector/).

**Table 3:** Oligonucleotides used in this study.

Name	Sequence (5′->3′)
AdsF	GGCGGGATCCAATGTCACTTACAGAAGAAAAACCTATTCGCC
AdsR	AGATCTGCGGCCGCTATATACTCATAGGATAAACGAG
tHMGRF	GACGTAAGGGATGACGCACAATC
tHMGRR	AGATCTGCGGCCGCCTATGTTGTTGTTGTTGTCGTTGTCGTTGC
XbaMluBamHF	CTAGACGAGGAATCGCTACGCGTCAG
XbaMluBamHR	GATCCTGACGCGTAGCGATTCCTCGT
MluStrepBamHF	CGCGTTGGTCTCATCCACAGTTCGAGAAGATG
MluStrepBamHR	GATCCATCTTCTCGAACTGTGGATGAGACCAA
MtXbaADSF	GCTCTAGAATGTTGTCACTACGTCAATCT
ADSStrepNotR	AGTTTAGCGGCCGCCTCTTCTCGAACTGTGGATGAGACCATCCACCGCCTATACTCATAGGATAAACGA
F2AMluF	GGCCGCAGACTTTGAATTTTGACCTTCTCAAGTTGGCGGGAGACGTGGAGTCCAACCCAGGGCCCACTAGTGGTGGCATATACGCGTA
F2AmluR	GATCTACGCGTATATGCCACCACTAGTGGGCCCTGGGTTGGACTCCACGTCTCCCGCCAACTTGAGAAGGTCAAAATTCAAAGTCTGC
MtXbaFDSF	GCTCTAGAGCACTAGTATGTTGTCACTACGTCAATCT
FDSStrepNotR	AGTTTAGCGGCCGCCCTTCTCGAACTGTGGATGAGACCACTTCTGCCTCTTGTAGATCT
T2AF	GGCCGCGAGGgCAGAGGAAGTCTTCTAACATGCGGTGACGTCGAGGAGAATCCTGG CCCAACGCGTA
T2AR	GATCTACGCGTTGGGCCAGGATTCTCCTCGACGTCACCGCATGTTAGAAGACTTCCTCTGCCCTCGC

*CYP71AV1* was obtained by digestion using BamHI and KpnI from a pYeDP60 vector provided by Anna-Margaretha Ryden. ImpactVector C3.1 (www.pri.wur.nl/UK/products/ImpactVector/) was adapted by insertion of a KpnI restriction site. For this purpose, an oligodimer was constructed as follows: two oligo's (GATCCATTTCGGTACCAATTAGC and GGCCGCTAATTG GTACCGAAATG) were paired and kinase-treated. The C3.1 vector containing a CaMV35S promoter and a RbcS1 terminator, was restricted with BamHI and NotI and the oligo-dimer was ligated into the vector. The resulting C3.1 + KpnI vector was digested with BamHI and KpnI. Gel-purified and BamHI and KpnI digested *CYP71AV1* gene was ligated into this vector, to yield 35S-CYP71AVI.

Several steps were required to construct the 2A vector with the genes: *ADS*, *FPS* and *tHMGR* (truncated 3-hydroxy-3-methylglutaryl-CoA reductase). *tHMGR* represents the truncated sequence of *HMGR* from *Arabidopsis thaliana* (accession number: J04537, 2195bp), *mADS* and *mFPS* represent the mitochondrial target sequence: Cox IV secretion signal from *S. cerevisiae* (GeneID: 852688) combined with the sequence of *ADS* from *Artemisia annua* (accession number: AAF61439, 1641bp) and the same Cox IV sequence fused to the *FPS2* sequence from *A. thaliana* (Genebank: NM_117823, 1026bp), respectively.

*tHMGR* was amplified using primers tHMGRF and tHMGRR (Table 3), and, after cleavage with BamHI and NotI, was cloned in vector pImpactVector3.1, which is a variant of pImpactVector1.1 but contains a CaMV35S promoter and a RbcS1 terminator (http://www.pri.wur.nl/UK/products/ImpactVector/). The resulting plasmid was cleaved using XbaI and BamHI restriction enzymes, and a duplex of oligonucleotides XbaMluBamHF and XbaMluBamHR was introduced. The resulting plasmid was cleaved with MluI and BamHI, and a linker of duplex MluStrepBamHF and MluStrepBamHR was introduced. The *mADS* cDNA was amplified using MtXbaADSF and ADSStrepNotR, and cloned into pImpactVector1.1. In this plasmid, the F2A peptide sequence was introduced as a linker of F2AMluF and F2AMluR in the NotI and BglII sites. The *FPS* cDNA was amplified using MtXbaFDSF and FDSStrepNotR, and cloned into pImpactVector1.1. In this plasmid, the T2A peptide sequence was introduced as a linker of T2AF and T2AR in the NotI and BglII sites. The *ADS* unit was cleaved from the pImpactVector1.1 by XbaI and MluI and introduced in the *HMGR* unit cleaved with the same restriction sites. Subsequently, the *FPS* unit was added to this construct by using SpeI and MluI restriction sites. The final construct was called 35S-mAmFH.

For individual expression, the *ADS* and *FDS* cDNAs were cloned into pImpactVector1.5, to fuse them to the RBC promoter and the CoxIV mitochondrial targeting sequence. The *tHMGR* gene was cloned into pImpactVector1.1 using BamHI and NotI restriction sites, to fuse it to the RBC promoter.

In order to construct a plant expression vector, the final constructs in pImpactVector were restricted using AscI and PacI. The fragments were ligated into the pBinPlus binary vector [32] between the right and left border of the T-DNA.

### *Agrobacterium* transformation

*Agrobacterium tumefaciens* AglI strain contains a disarmed Ti plasmid that provides the vir gene functions. It harbors rifampicillin and carbenicillin chromosomal resistance genes [33]. The binary vector was introduced into AglI by electrotransformation.

### Transient expression in leaves of *Nicotiana benthamiana*

*Agrobacterium* strains were grown at 28°C at 220 rpm for 24 hours in LB media with kanamycin (50 mg/L) and rifampicillin (34 mg/L). Cells were harvested by centrifugation for 20 min at 4000 g and 20°C and then resuspended in 10 mM MES buffer containing 10 mM MgCl_2_ and 100 µM acetosyringone (4′-hydroxy-3′,5′-dimethoxyacetophenone, Sigma) to a final OD600 of 0.5, followed by incubation at room temperature and 50 rpm for 150 minutes. For co-infiltration, equal volumes of the *Agrobacterium* strains were mixed. In all experiments, an *Agrobacterium* strain harbouring a gene encoding the TBSV P19 protein was added to maximize protein production by suppression of gene silencing [34]. *Nicotiana benthamiana* plants were grown from seeds on soil in a greenhouse with 16 h light at 28°C (16 h)/25°C (8 h). Strain mixtures were infiltrated into leaves of four-week-old *N. benthamiana* plants using a 1 mL syringe. The bacteria were slowly injected into the abaxial side of the leaf. The plants were grown under greenhouse conditions until further analysis.

### Headspace analysis and GC-MS thermodesorption

Steel sorbent cartridges (89 mm ×6.4 mm O.D.; Markes) containing 200 mg Tenax TA 20/35 for collection of volatiles were conditioned for 40 min at 280°C under a nitrogen flow of 20 psi using a TC-20 multi-tube conditioner and were kept airtight with brass caps until use. Freshly collected *N. benthamiana* leaves were placed on water enclosed in a glass container. Air was sucked through the containers with a flow rate of 90 ml.min^−1^ for 24 h through the Tenax cartridges to trap plant-produced volatiles. Incomming air was purified with a second Tenax cartridge. Before GC-MS analysis, the cartridges were dried for 15 min at room temperature with a nitrogen flow of 20 psi.

Headspace samples were analyzed with a Thermo TraceGC Ultra connected to a Thermo TraceDSQ quadrupole mass spectrometer (Thermo Fisher Scientific, Waltham). Before thermodesorption, traps were flushed with helium at 50 ml min^−1^ for 2 min to remove moisture and oxygen. After flushing the collected volatiles were desorbed from the Tenax traps at 220°C (Ultra; Markes, Llantrisant) for 5 min with a helium flow of 50 ml.min^−1^. The released compounds were focused on an electrically cooled sorbent trap (Unity; Markes, Llantrisant) at a temperature of 5°C. Volatiles were injected on the analytical column (ZB-5MSI, 30 m ×0.25 mm ID, 1.0 µm – film thickness, Zebron, Phenomenex) in splitless mode by ballistic heating of the cold trap to 250°C for 3 min. The temperature program started at 40°C (3 min hold) and rose 10°C.min^−1^ to 280°C (2 min hold). The column effluent was ionised by electron impact (EI) ionisation at 70 eV. Mass scanning was done from 33 to 280 m/z with a scan time of 4.2 scans.s^−1^. The eluted compounds were identified using Xcalibur software (Thermo, Waltham) by comparing the mass spectra with those of authentic reference standards.

### GC-MS analysis of extracts

Compounds accumulated in the leaves were analysed by snapfreezing and grinding 500 mg infiltrated leaf from each treatment in liquid nitrogen and extraction with 2 ml ethyl acetate. The extracts were prepared by brief vortexing and sonication for 15 min. Then the extracts were centrifuged for 15 min at 3500 rpm and dehydrated using anhydrous Na_2_SO_4_. For analysis of artemisinic acid, samples were methylated using diazomethane. The samples were analyzed by GC-MS using a gas chromatograph (5890 series II, Hewlett-Packard) equipped with a 30 m ×0.25 mm, 0.25 mm film thickness column (5MS, Hewlett-Packard) and a mass-selective detector (model 5972A, Hewlett-Packard). For analysis, 1 µl was injected, and the column temperature was increased from 45°C to 280°C in 20 minutes. Standards of amorpha-4,11-diene, artemisinic acid, dihydroartemisinic acid, artemisinic alcohol, dihydroartemisinic alcohol, artemisinic aldehyde and dihydroartemisinic aldehyde [4] were injected for reference.

### Analysis by LC-QTOF-MS/MS

Non-volatile compounds were analysed using a protocol for untargeted metabolomics of plant tissues [35]. In brief, 500 mg infiltrated leaf from each treatment was ground in liquid nitrogen and extracted with 2 ml methanol:formic acid (1000∶1, v/v). The extracts were prepared by brief vortexing and sonication for 15 min. Then the extracts were centrifuged for 5 min at 13,000 rpm and filtered through 0.2 µm inorganic membrane filters (RC4, Sartorius, Germany). Liquid chromatography, coupled to quadrupole time-of-flight mass spectrometry (LC–QTOF-MS) was performed using a Waters Alliance 2795 HPLC connected to a Waters 2996 PDA detector and subsequently a QTOF Ultima V4.00.00 mass spectrometer (Waters, MS technologies, UK) operating in negative ionization mode. The column used was an analytical column (Luna 3 µ C18/2 100A; 2.0×150 mm; Phenomenex, USA) attached to a C18 pre-column (2.0×4 mm; AJO-4286; Phenomenex, USA). Degassed eluent A (ultra pure water:formic acid (1000∶1,v/v) ) and eluent B (acetonitril:formic acid (1000∶1,v/v) ) were used at 0.19 ml.min^−1^. The gradient started at 5% B and increased linearly to 35% B in 45 min, after which the column was washed and equilibrated for 15 min before the next injection. The injection volume was 5 µl. The MS-MS measurements were done with collision energies of 10, 15, 25, 35 and 50 eV. Leucine enkaphalin ([M-H]^−^ = 554.2620) was used as a lock mass for on-line accurate mass correction.

### Analysis of LC-MS data and identification of AA-di-hexose

LC-MS data were analysed using MassLynx 4.0 (Waters). The data were first processed using MetAlign version 1.0 (www.metAlign.nl) for baseline correction, noise elimination and subsequent spectral data alignment [35]. The processing parameters of MetAlign were set to analyze from scan number 60 to 2300 with a maximum amplitude of 35000. Mass signals below 10 times the local noise were filtered out and the resulting data matrix contained intensity values (calculated as peak heights) for 6645 mass signals aligned across all samples. The mass intensity data were 2log-transformed. Mass signal intensities obtained from transgenic plants and empty vector control plants were compared using the Student's t-test. Masses with a significant (p<0.05) intensity change of at least 2-fold were verified manually in the original chromatograms. Mass-directed LC-MS/MS analysis was done on differential compounds with signal intensities higher than 500 ion counts per scan.

### Compound isolation by LC-FC/SPE

First purification was carried out on an Agilent 1200 system (Agilent Technology, SantaClara (CA) USA) equipped with an analytical scale fraction collector, using Zorbax Eclipse XDB-C18, 5 µm, 4.6×150 mm column (Agilent). at an isocratic concentration of 35% acetonitrile and 0.1% formic acid. In this purification step the compound of interest is separated from most of the other compounds in the sample. A second purification was carried out on the same system, now using a Prospekt 2 and general purpose (GP) cartridges (SPARK Holland) to trap the compound. The material was dried for at least 40 minutes under N_2_ air flow. Elution was performed using 600 uL of deuterated acetonitrile (99.96%, Eurisotop).

### NMR spectroscopy

NMR analysis was performed at Spinnovations BV (Nijmegen) on a Bruker Avance III 500 MHz spectrometer equipped with a 5-mm CPTCI cryo probe (^1^H-^13^C/^15^N/^2^H + Z-gradients) operating at 303 K. The structure identification of artemisinic acid as a reference compound is based on a 1D ^1^H, ^1^H-^1^H-DQF-COSY, ^1^H-^1^H-TOCSY, ^1^H-^1^H-NOESY, ^1^H-^13^C-HSQC and ^1^H-^13^C-HMBC spectra. The structure identification of the purified compound is based on the same selection of NMR experiments with the exception of the ^1^H-^13^C-HMBC spectrum due to the limited amount of material. The proton and carbon chemical shifts were referenced to the internal reference TMS (proton, δ = 0.00 ppm; carbon, δ = 0.00 ppm). The data were processed using Topspin 2.1 pl5.

### Glycosidase treatment

To identify glycosylated metabolites, 0.5 µg of purified AA-dihexoside was tested in the appropriate buffer system with 0.1 unit of yeast α-glucosidase (Sigma; 50 mM potassium phosphate pH = 7.5), almond β-glucosidase (Sigma; 50 mM sodium acetate pH = 5.6) or jack bean β-galactosidase (50 mM citrate pH = 4+0.01% BSA), and incubated for 3 hrs at 37°C. Samples were analyzed using LC-MS and GC-MS as described above.

To quantify AA-diglucoside, 200 mg infiltrated leaf material from each treatment was ground in liquid nitrogen and extracted with 1 ml citrate phosphate buffer, pH 5.4. The extracts were prepared by brief vortexing and sonication for 15 min. 200 µl of ViscozymL (Sigma) was added and the sample again vortexed. The mixture was incubated overnight at 37°C, and subsequently extracted three times with 1 ml of ethyl acetate. Extracts were dehydrated using Na_2_SO_4_, concentrated to approximately 250 µl and methylated using diazomethane. An internal standard, *cis*-nerolidol, was used to quantify the products. Artemisinic acid, when present in a plant extract, was not fully methylated by diazomethane. Therefore, both unmethylated artemisinic acid (selective mass m/z 234, Rt = 17.69; Fig. S5) and methylated artemisinic acid (total ion count, Rt 16.98 min; Fig. S6) standards were used for quantification. Samples were analyzed using GC-MS as described above.

## Supporting Information

Table S1 Mass signals significantly different between samples infiltrated with 35S-mAmFH-2A + 35S-CYP71AVI and 35S-mAmFH-2A. Data are sorted by retention time.
(1.04 MB DOC)

Figure S1 Mass spectrum (negative mode) of artemisinic acid-12-β-diglucoside, showing a base-peak mass ([M-H]), a formic acid adduct ([M-H + H2CO2]) and a dimeric mass ([2M-H]).
(0.01 MB PDF)

Figure S2 MSMS mass spectrum, showing collision mass fragments of artemisinic acid-12-β-diglucoside (m/z 557.24; [M-H]).
(0.01 MB PDF)

Figure S3 The effect of β-glucosidase on artemisinic acid-12-β-diglucoside. (A) Total ion count mass chromatograms and (B) chromatograms at m/z  =  791.5. Represented are chromatograms of artemisinic acid-12-β-diglucoside treated with control (thin line) or β-glucosidase (bold line). The peak at Rt  =  28.0 min represents artemisinic acid-12-β-diglucoside, the peak at Rt  =  53.2 min represents artemisinic acid monoglucoside.
(0.02 MB PDF)

Figure S4 GC-MS chromatogram (total ion count) of viscozym-treated extracts of N. benthamiana leaves infiltrated with 35S-mAmFH-2A (bottom) or with 35S-mAmFH-2A + 35S-CYP71AV1 (top). Indicated peaks have been further analyzed.
(0.01 MB PDF)

Figure S5 GC-MS chromatogram (m/z 234) of viscozym-treated extracts of N. benthamiana leaves infiltrated with 35S-mAmFH-2A (bottom), with 35S-mAmFH-2A + 35S-CYP71AV1 (middle), or artemisinic acid standard (top). Mass spectra of artemisinic acid and the artemisinic acid produced in tobacco are shown.
(0.04 MB PDF)

Figure S6 GC-MS chromatogram (m/z 248) of viscozym-treated extracts of N. benthamiana leaves infiltrated with 35S-mAmFH-2A (bottom), with 35S-mAmFH-2A + 35S-CYP71AV1 (middle), or artemisinic acid standard (top). Mass spectra of methylation products of artemisinic acid and the artemisinic acid produced in tobacco are shown.
(0.03 MB PDF)
