# Supplementary material for: Nicotiana benthamiana as a Production Platform for Artemisinin Precursors
Source: PLoS One. 2010 Dec 3;5(12):e14222. doi: 10.1371/journal.pone.0014222 (PMC2997059; doi:10.1371/journal.pone.0014222)
Supplement: Table S1 — Mass signals significantly different between samples infiltrated with 35S-mAmFH-2A + 35S-CYP71AVI and 35S-mAmFH-2A. Data are sorted by retention time. (1.04 MB DOC) [file pone.0014222.s001.doc]

Suppl. Table 1: mass signals significantly different between samples infiltrated with 35S-mAmFH-2A + 35S-CYP71AVI and 35S-mAmFH-2A. Data are sorted by retention time.
