# Supplementary material for: Nicotiana benthamiana as a Production Platform for Artemisinin Precursors
Source: PLoS One. 2010 Dec 3;5(12):e14222. doi: 10.1371/journal.pone.0014222 (PMC2997059; doi:10.1371/journal.pone.0014222)
Supplement: Figure S1 — Mass spectrum (negative mode) of artemisinic acid-12-β-diglucoside, showing a base-peak mass ([M-H]), a formic acid adduct ([M-H + H2CO2]) and a dimeric mass ([2M-H]). (0.01 MB PDF) [file pone.0014222.s002.pdf]

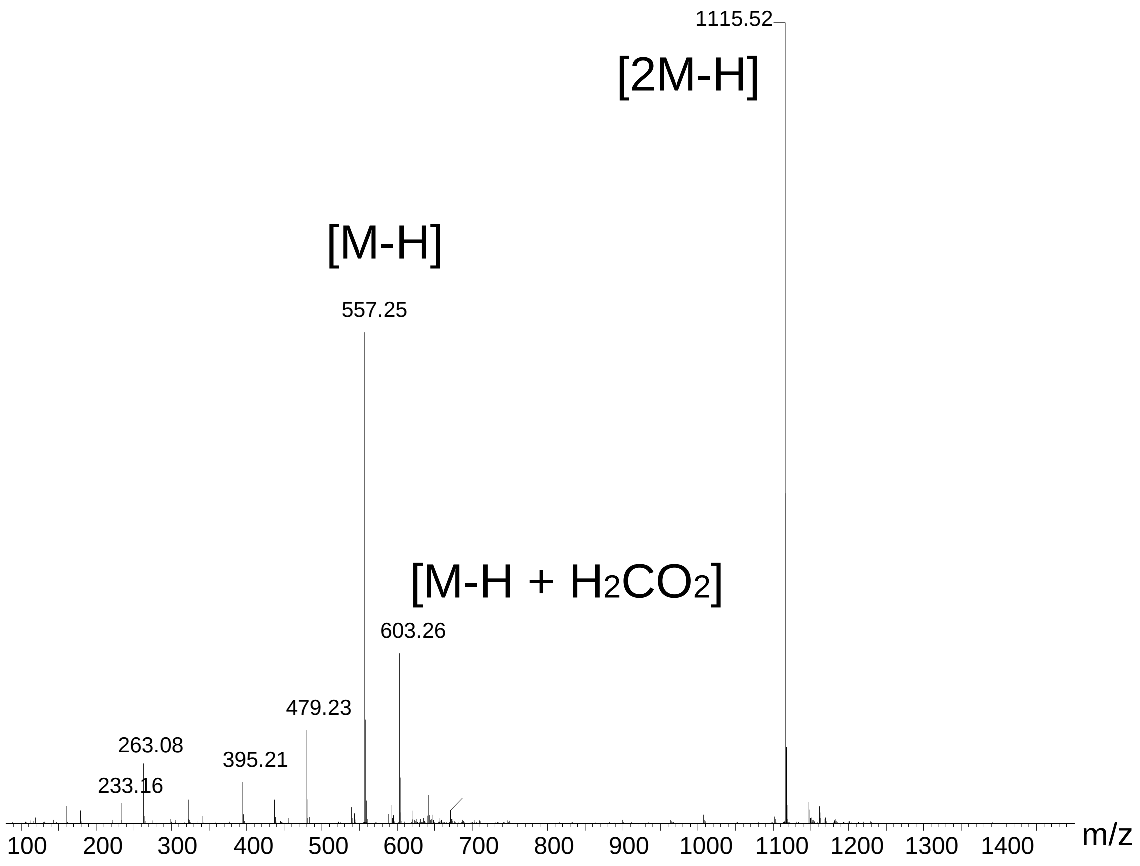

Figure S1: mass spectrum (negative mode) of artemisinic acid-12-β-diglucoside, showing a base-peak mass ([M-H]), a formic acid adduct ([M-H + H2CO2]) and a dimeric mass ([2M-H]).
