# Supplementary material for: Nicotiana benthamiana as a Production Platform for Artemisinin Precursors
Source: PLoS One. 2010 Dec 3;5(12):e14222. doi: 10.1371/journal.pone.0014222 (PMC2997059; doi:10.1371/journal.pone.0014222)
Supplement: Figure S2 — MSMS mass spectrum, showing collision mass fragments of artemisinic acid-12-β-diglucoside (m/z 557.24; [M-H]). (0.01 MB PDF) [file pone.0014222.s003.pdf]

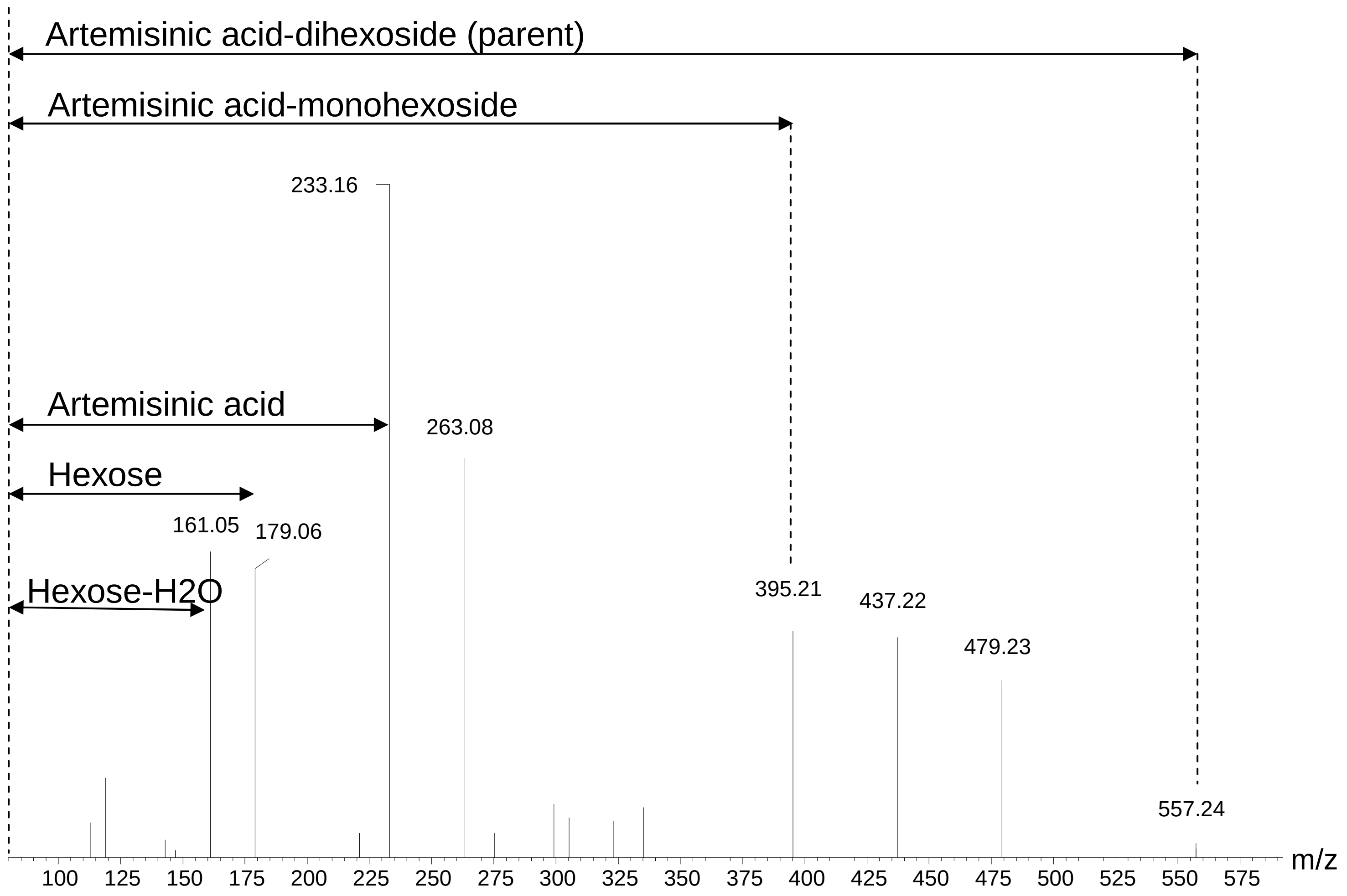

Figure S2: MSMS mass spectrum, showing collision mass fragments of artemisinic acid-12-β-diglucoside (m/z 557.24; [M-H]).
