# Supplementary material for: Nicotiana benthamiana as a Production Platform for Artemisinin Precursors
Source: PLoS One. 2010 Dec 3;5(12):e14222. doi: 10.1371/journal.pone.0014222 (PMC2997059; doi:10.1371/journal.pone.0014222)
Supplement: Figure S3 — The effect of β-glucosidase on artemisinic acid-12-β-diglucoside. (A) Total ion count mass chromatograms and (B) chromatograms at m/z = 791.5. Represented are chromatograms of artemisinic acid-12-β-diglucoside treated with control (thin line) or β-glucosidase (bold line). The peak at Rt = 28.0 min represents artemisinic acid-12-β-diglucoside, the peak at Rt = 53.2 min represents artemisinic acid monoglucoside. (0.02 MB PDF) [file pone.0014222.s004.pdf]

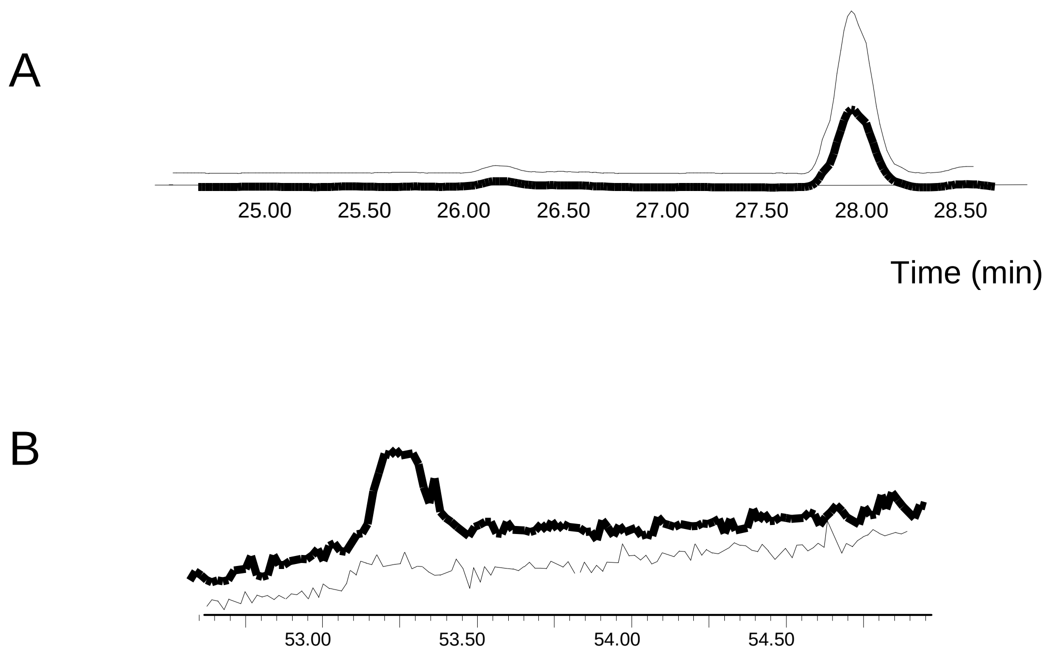

Figure S3: The effect of β-glucosidase on artemisinic acid-12-β-diglucoside. (A) Total ion count mass chromatograms and (B) chromatograms at *m/z* = 791.5. Represented are chromatograms of artemisinic acid-12-β-diglucoside treated with control (thin line) or β-glucosidase (bold line). The peak at Rt = 28.0 min represents artemisinic acid-12-β-diglucoside, the peak at Rt = 53.2 min represents artemisinic acid monoglucoside.
