# Supplementary material for: Nicotiana benthamiana as a Production Platform for Artemisinin Precursors
Source: PLoS One. 2010 Dec 3;5(12):e14222. doi: 10.1371/journal.pone.0014222 (PMC2997059; doi:10.1371/journal.pone.0014222)
Supplement: Figure S5 — GC-MS chromatogram (m/z 234) of viscozym-treated extracts of N. benthamiana leaves infiltrated with 35S-mAmFH-2A (bottom), with 35S-mAmFH-2A + 35S-CYP71AV1 (middle), or artemisinic acid standard (top). Mass spectra of artemisinic acid and the artemisinic acid produced in tobacco are shown. (0.04 MB PDF) [file pone.0014222.s006.pdf]

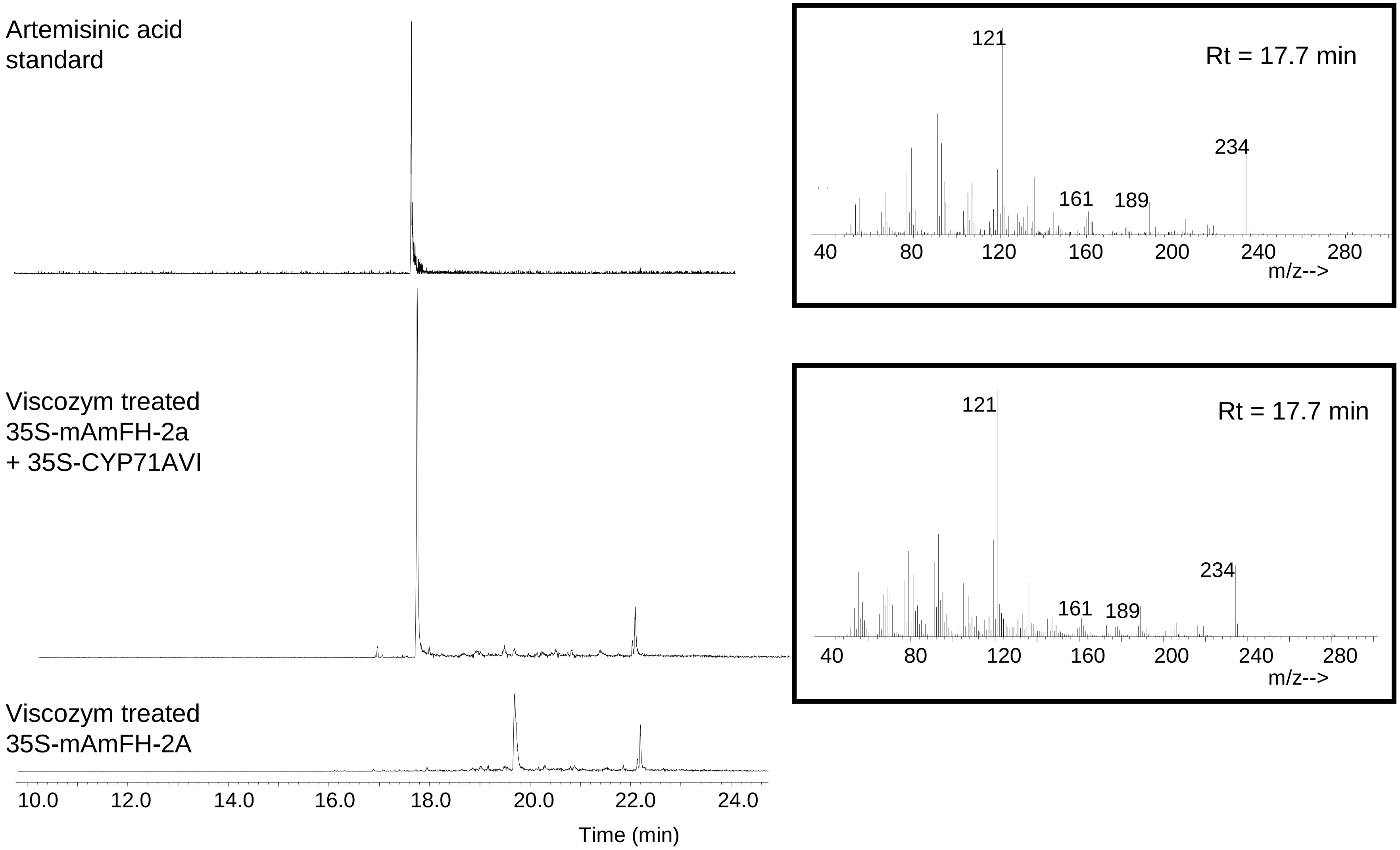

Figure S5 GC-MS chromatogram (m/z 234) of viscozym-treated extracts of *N. benthamiana* leaves infiltrated with 35S-mAmFH-2A (bottom), with 35S-mAmFH-2A + 35S-CYP71AV1 (middle), or artemisinic acid standard (top). Mass spectra of artemisinic acid and the artemisinic acid produced in tobacco are shown.
